# *E. coli* O157:H7 evades CD8^+^ T cell immunity through T3SS-dependent suppression and limited MHCI presentation of translocated proteins

**DOI:** 10.1093/discim/kyag017

**Published:** 2026-09-09

**Authors:** Amany Hassan, Annalisa Nicastri, Deepali Vasoya, Joana Alves, Morten Nielsen, David L Gally, Nicola Ternette, Alexander Corbishley, Timothy K Connelley

**Affiliations:** Department of Animal Medicine, The Faculty of Veterinary Medicine, The University of Alexandria, Alexandria, Egypt; The Royal (Dick) School of Veterinary Studies and Roslin Institute, University of Edinburgh, Midlothian, UK; Department of Veterinary Microbiology and Pathology, Washington State University, Pullman, WA, USA; The Jenner Institute, Nuffield Department of Medicine, The University of Oxford, Oxford, UK; UK Dementia Research Institute at University of Edinburgh, Edinburgh Bioquarter, Edinburgh, UK; The Royal (Dick) School of Veterinary Studies and Roslin Institute, University of Edinburgh, Midlothian, UK; Department of Health Technology, Technical University of Denmark, Lyngby, Denmark; The Royal (Dick) School of Veterinary Studies and Roslin Institute, University of Edinburgh, Midlothian, UK; The Jenner Institute, Nuffield Department of Medicine, The University of Oxford, Oxford, UK; Faculty of Life Sciences, University of Dundee, Dundee, UK; The Royal (Dick) School of Veterinary Studies and Roslin Institute, University of Edinburgh, Midlothian, UK; The Royal (Dick) School of Veterinary Studies and Roslin Institute, University of Edinburgh, Midlothian, UK

**Keywords:** Enterohemorrhagic Escherichia coli, T3SS, immunopeptidomics, evasion, CD8^+^, T-cell, immunity, vaccine

## Abstract

**Introduction:**

Enterohemorrhagic *Escherichia coli* (EHEC) O157:H7 causes severe disease in humans, but colonizes cattle asymptomatically. Our previous research showed a Th1-biased mucosal immune response in colonized cattle, with antigen-specific CD8^+^ T cell proliferation. EHEC O157:H7 colonizes the bovine gastrointestinal tract, a process that requires a type 3 secretion system and injection of a cocktail of ‘effector’ proteins into epithelial cells, resulting in tight bacterial/epithelial attachment. Mathematical modeling indicates EHEC O157:H7 effector proteins have reduced MHC Class I (MHCI) ligand density, potentially an adaptation to evade CD8^+^ T cell immunity when injected into host cells.

**Methods:**

In this study, immunopeptidomics was applied to study the presentation of *E. coli* peptides on MHCI molecules using an *in vitro* bovine epithelial cell colonization model. peptide elution from bovine epithelial cells colonized with *E. coli* O157:H7.

**Results:**

Immunopeptidomic analysis of bovine epithelial cells colonized with *E. coli* O157:H7 experimentally confirmed a scarcity of MHCI-presented peptides from secreted bacterial effector proteins. Unexpectedly, two MHCI ligands were identified from intimin, a bacterial outer membrane protein crucial for cattle colonization, with one ligand overlapping a known CD4^+^ T cell epitope. Using a bovine epithelial cell line transfected with the BoLA-1*023:01 MHCI allele, we showed that *E. coli* O157:H7 suppresses antigen-specific CD8^+^ T-cell activation.

**Conclusion:**

The study results highlight the importance of engaging both CD4^+^ and CD8^+^ T cell responses for rational vaccine design, an area not traditionally emphasized for extracellular pathogens such as *E. coli* O157:H7. By combining immunopeptidomics and bespoke *in silico* prediction algorithms, we provide a foundation for next-generation vaccines aimed at more effectively clearing persistent *E. coli* O157:H7 infections in cattle and reducing the risk of zoonotic transmission.

## Graphical Abstract

**Graphical Abstract:**
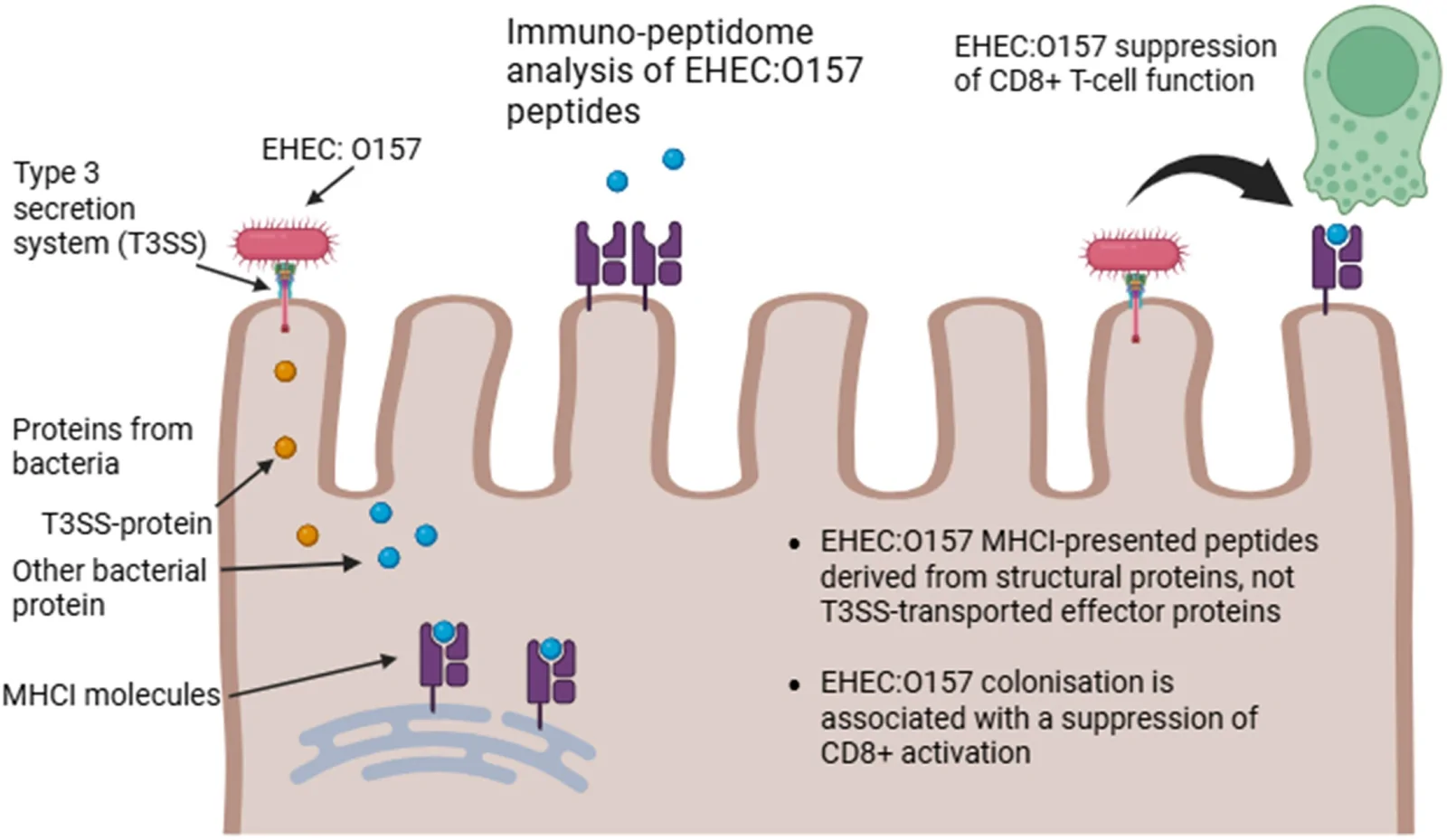

## Introduction

Enterohaemorrhagic *Escherichia coli* (EHEC) O157:H7 is a global foodborne pathogen that poses a significant risk to human health. Infections can range from asymptomatic to diarrhea and life-threatening systemic complications, including kidney failure and brain damage. The severity of these pathologies is attributed to the production of Shiga toxins, which affect endothelial cells lining capillaries that are positive for the Gb3 (CD77) receptor [1–3]. While clinical disease primarily affects humans, cattle serve as the main reservoir of infection, where the bacteria asymptomatically colonize the bovine gastrointestinal tract [4–7]. This colonization requires a type 3 secretion system (T3SS), which transports effector proteins into host cells [8, 9]. These effectors play a crucial role, triggering downstream signaling events that enable bacterial persistence in the host. One facet of effector protein activity is the rearrangement of the actin cytoskeleton, which enables intimate binding between the bacterium and the host cell [10–12]. In particular, the translocated intimin receptor (Tir) is a bacterial effector that once injected, integrates into the host cell membrane and acts as a receptor for the bacterial outer membrane protein, intimin. Tir is among ∼40 different proteins that are translocated into epithelial cells through the bacterial T3SS [13, 14].

The delivery of bacterial effectors into the host cell cytosol makes them potentially available to the antigen processing and presentation pathway associated with MHC Class I (MHCI) molecules. However, mathematical modeling studies indicate that whilst injected *E. coli* effector proteins, including Tir, are susceptible to proteasomal cleavage, their TAP (transporter associated with antigen processing) and MHCI binding scores are reduced compared to structural bacterial proteins [15]. This suggests that similar to viral proteins [16], injected bacterial effector proteins have undergone evolutionary selection for reduced MHCI ligand densities. That said, our previous research has revealed evidence of EHEC O157:H7 antigen-specific CD4^+^ and CD8^+^ T-cell responses at the terminal rectum of colonized calves [17]. Taken together, these data indicate that EHEC O157:H7 colonization induces a CD8^+^ T-cell response, and, in order to mitigate against this, the bacteria may have evolved mechanisms to reduce MHCI presentation of injected effector proteins so as to facilitate colonization in the reservoir host.

This has relevance to vaccine development, given the variable efficacy of existing cattle vaccines against EHEC O157:H7 [18–21]. These vaccines elicit mucosal antibody responses that are posited to reduce initial bacterial adherence to the apical epithelial surface [22–24]; however, their efficacy in reducing bacterial shedding remains variable [23, 25, 26]. A deeper understanding of the role of the adaptive cellular immune response during EHEC O157:H7 clearance from the mucosal epithelium would help inform the generation of new vaccines designed to elicit strong adaptive cellular immune responses and hence potentially reduce the level and duration of bacterial shedding following colonization.

This study pursued two objectives. First, it sought to employ immunopeptidomics to evaluate the ability of bovine epithelial cells to present *E. coli*-derived peptides on MHCI. Second, it aimed to establish an *in vitro* bovine system designed to explore the interactions between bovine epithelial [embryonic bovine lung (EBL)] cells colonized with *E. coli* O157:H7 and bovine CD8^+^ T cells. Our immunopeptidomic studies characterized thousands of MHCI ligands presented by infected EBL cells, with a paucity in the presentation of bacterial-derived peptides. A notable feature of the bacterial immunopeptidome was the presentation of two MHCI ligands derived from intimin, one of which overlaps with a previously published CD4^+^ T cell epitope. We also demonstrated that *E. coli* O157:H7 colonization inhibited antigen-specific activation of bovine CD8^+^ T cells, supporting the hypothesis that *E. coli* O157:H7 has developed strategies to help evade CD8^+^ T-cell immune responses.

These findings highlight a role for CD8^+^ T cells in the clearance of the extracellular bacterium *E. coli* O157:H7, underscoring the importance of considering both arms of adaptive cellular immunity, as well as antibodies, in vaccine design. The combination of direct demonstration of MHCI presentation of *E. coli* O157:H7 peptides by epithelial cells through immunopeptidomic analysis and evidence that these bacteria have the capacity to suppress CD8^+^ T cell responses supports targeting of CD8^+^ T-cell responses in cattle vaccines aimed at reducing the incidence and excretion of *E. coli* O157:H7 to limit onward zoonotic transmission.

## Materials and methods

### Cell lines

EBL cells (RRID: CVCL_2028) were cultured in RPMI 1640-HEPES modified medium (Sigma-Aldrich, Gillingham, UK) containing 10% heat-inactivated fetal calf serum (HI-FCS), 2 mM L-glutamine, 200 units/ml penicillin, 250 µg/ml streptomycin (PSG), and 1 mM beta-mercaptoethanol (β-ME) (referred to as standard medium) at 37°C with 5% CO_2_. Cells were passaged every 2–3 days upon reaching 80–100% confluence. No bovine intestinal cell lines were available for the study. Previous studies had confirmed that *E. coli* O157:H7 can successfully colonize EBL cells as a bovine epithelial cell model in which clear attaching and effacing lesions are formed as a consequence of effector protein translocation by the T3SS [27].

### Bacterial cultures

*Escherichia coli* O157:H7 wild-type strain (ZAP193, human origin, Shiga toxin negative NCTC12900) and an isogenic mutant with a non-functional T3SS, due to deletion of the basal apparatus ATPase *escN* (Δ*escN*, ZAP2138), were utilized in the study [28]. ZAP2138 was demonstrated to be non-colonizing in work completed as part of the PhD thesis of the first author (http://dx.doi.org/10.7488/era/1460)—Figure 2.6. For colonization studies, a single colony was inoculated into Lysogeny Broth (LB) and incubated at 37°C, 200 rpm overnight. A 1:100 dilution was made into minimal essential medium (Eagle) with HEPES modification (MEM-HEPES, Sigma-Aldrich) supplemented with 0.1% glucose and 0.1 nM Fe(NO_3_)_3_ (Sigma-Aldrich) (referred to as cMEM-HEPES), and incubated overnight at 37°C at 200 rpm. Bacterial culture supernatants were generated using the same culture conditions (but with an OD_600_ of 0.8), centrifuged at 12 000 rpm, and the resulting supernatants filtered through 0.2 μm low-protein binding filters (Millipore) and stored at −20°C.

### Optimization of colonization conditions

EBL cells were cultured on collagen-coated coverslips (MIC-3306, SLS, UK) in 12-well plates until they reached ∼80% confluence (∼2.5 × 10^5^ cells/well). The experimental conditions were established in triplicate, with a matrix of EBL cultured with bacteria at an initial multiplicity of infection (MOI) of either 0, 10, or 100, for 3, 4, 6, or 7 hours at 37°C with 5% CO_2_. One hour prior to the experiment, the EBL culture medium was replaced with antibiotic-free cMEM-HEPES (Sigma-Aldrich). For co-culture periods of greater than 3 hours, phosphate-buffered saline (PBS) washes were performed at 3-hour intervals to remove bacteria growing in the culture medium that had not colonized the EBL cells. At the end of the culture period, cells were fixed using a 2% formaldehyde solution in PBS (Thermo Scientific). Cells were permeabilized with 0.1% Triton X-100 (Sigma-Aldrich), stained sequentially with anti-*E. coli* O157 antibody (rabbit polyclonal antibody clone, Abcam), Alexa Fluor 568-conjugated goat anti-rabbit polyvalent antibody (Abcam), FITC-phalloidin (for staining F-actin; Sigma-Aldrich), and Hoechst-33342 (for nuclei staining; Sigma-Aldrich). Between staining steps, cells were washed with PBS. Coverslips were mounted onto glass slides using Prolong-Diamond Antifade mounting (Invitrogen) and imaging was conducted with a Leica DMLB fluorescent microscope.

### Immunopeptidomic analysis

1 × 10^9^ EBL cells were cultured for 3 hours with an MOI of 100 of either ZAP193, ZAP2138, or with no bacteria (uninfected control) as described above, harvested using non-enzymatic cell dissociation solution (Gibco) and BoLA-I associated peptide purification was performed as previously described [29]. In brief, samples were resuspended in 20 μl of loading buffer and analyzed in a nano UPLC Ultimate 3000 system coupled to a Q Extractive HF mass spectrometer (Thermo Scientific). Peptides were separated in a 75 μm × 50 cm PepMap C18 column and resolved in a 60 min gradient of 5–30% buffer B in buffer A at a flow rate of 250 nL/min (∼600 bar). Peptides were introduced into the mass spectrometer using a nano Easy Spray source (Thermo Scientific). Subsequent isolation and higher energy C trap dissociation was induced in the 20 most abundant ions per full MS scan with an accumulation time of 128 min and an isolation width of 1.0 Da. All fragmented precursor ions were actively excluded from repeated selection for 30 s. MS spectra were searched simultaneously against a database comprising downloaded TrEMBL entries for the *Bos taurus* proteome (38 000 entries) concatenated with the ZAP193 proteome (5100 proteins) based on the annotated DNA-seq data of *E. coli* O157:H7 EDL933 (taxid: 562, nr database-BLASTP; see Supplementary Data S1) using PEAKS v7.5. A value of 15 was used as the threshold for peptide-spectrum score values (−10lgP), resulting in an estimated false discovery rate (FDR) below 5%. Several standard preliminary parsing steps were completed for the *E. coli* peptide datasets: (i) all peptides that matched both *Bos taurus* and *E. coli* proteins were considered to be ambiguous and were removed; (ii) *E. coli* peptides were checked for isoleucine/leucine switches and any peptides that could have consequently been misannotated *Bos taurus* peptides were removed; and (iii) any *E. coli* peptides found in the uninfected control were removed from all datasets as they likely represented unannotated bovine peptides.

### BoLA-I genotyping

RNA was extracted from EBL cells using the RNeasy Plus Mini Kit (Qiagen, Hilden, Germany), and cDNA was synthesized using the iScript cDNA Synthesis Kit (BIO-RAD, Hertfordshire, UK), both according to the manufacturers’ instructions. PCR amplification, sample preparation, sequencing, and subsequent bioinformatic processing of the data was completed as described previously [30, 31].

### Generation and selection of BoLA-1*023:01^+^ EBL cells

ILA89, CC218 (both specific for polymorphic bovine MHCI epitopes present on the BoLA-1*023:01 allele), and ILA88 (specific for a pan-bovine MHCI epitope) were used to determine if these antibodies could be used in combination to subsequently confirm expression of BoLA-1*023:01 when transduced into the EBL cell line. All primary antibodies were kindly provided by Prof. Ivan Morrison (The Roslin Institute, University of Edinburgh) and used in sequential staining with a secondary anti-murine pan IgG Alexa-647-conjugated antibody (Life Technologies, Paisley, UK). A no primary antibody control was included in all experiments. Staining was for 30 min at 4°C in a 5% HI-FCS/0.02% sodium azide/PBS buffer which was also used for washing between staining. Following staining, cells were analyzed using a FACS LSR Fortessa flow cytometer (BD Biosciences, San Jose, USA) and FlowJo v10.0.7 (FlowJo, Ashland, USA).

RNA from a BoLA-1*023:01-transfected P815 cell line (kindly provided by Prof. Ivan Morrison [32]) was extracted and cDNA synthesized as described above. Near full length coding BoLA-1*023:01 amplicons were generated with designed primers that incorporated SalI and HindIII digestion sites (Forward BoLA-1*023:01: TAAGCAAAGCTTATGGGGCCGCGAACC, and reverse BoLA-1*023:01: TAAGCAGTCGACGACTCACACTTTAGGAACCG). PCR reactions were conducted as previously described [29, 30], except for an annealing temperature of 70°C for 20 s. Following sequence confirmation, the BoLA-1*023:01 amplicon was cloned into a green fluorescent protein (GFP) turbo cloning vector (kindly provided by Yolanda Corripio-Miyar [33]) and used to transform DH5α competent *E. coli* (Invitrogen, Renfrew, UK). Transformants were cultured on selective LB agar containing kanamycin (50 µg/ml) and the presence of the BoLA-1*023:01 amplicon was confirmed by PCR. Selected colonies were cultured overnight in LB medium with kanamycin (50 µg/ml), followed by plasmid purification using the E.Z.N.A. Plasmid Mini Kit (Omega Bio-tek, Norross, GA, USA) according to the manufacturer’s instructions. Following sequence confirmation, EBL cells were transfected with BoLA-1*023:01^+^ by plasmid electroporation. Briefly, 1 × 10^6^ cells were mixed with 1 μg of plasmid DNA and 100 µl of Nucleofector Solution (Amexa Kit V) and electroporated using a Nucleofector 2D device (Lonza, Basel, Switzerland), program T-030. After electroporation, transfected cells were maintained in selective culture media supplemented with 500 μg/ml neomycin (G418, Sigma-Aldrich) for one week. Further selection used flow cytometry sorting of BoLA-1*023:01^+^ EBL cells following sequential staining with ILA89 and anti-IgG Alexa-647 conjugated antibodies (positive selection of GFP+/Alexa-647+ cells) using a FACS Aria IIIu-cell sorter (BD Biosciences, San Jose, CA, USA). Sorted cells were then maintained in selective medium.

### Antigen-specific CD8^+^ T cell stimulation by transduced BoLA-1*023:01^+^ EBL cells

495-CD8^+^ T-cells, specific for the BoLA-1*023:01-restricted Tp9­_67–75_ (AKFPGMKKS) epitope from *Theileria parva* Muguga [34], were kindly provided by Prof. Ivan Morrison. 495-CD8^+^ T-cells were maintained by weekly stimulation with γ-irradiated 2229TpM (a BoLA-1*023:01 expressing *T. parva*-infected cell line) and culture in RPMI 1640 media supplemented with 10% HI-FCS/β-ME/PSG and 100 IU/ml of recombinant human IL-2 (Thermo Scientific) using previously published protocols [35]. 2229TpM and 592TpM (a non-BoLA-1*023:01-expressing *T. parva*-infected cell line) were maintained by culture in RPMI 1640 media supplemented with 10% HI-FCS/β-ME/PSG (standard media) and passaged every 2–3 days.

In the IFN-γ release assays, target (transformed EBL) cells were seeded at 5 × 10^4^ cells/well in wells of a 96-well plates and pulsed (1 hour incubation in a humidified 5% CO_2_ atmosphere at 37°C) with either Tp9­_67–75_ (target epitope) or Tp2_98–106_ (an irrelevant peptide used as a negative control) [33] in a 3-fold serial dilution series ranging from 2 × 10^−6^ to 3 × 10^−11^ M (in triplicate for each condition). Cells were then washed three times using 150 μl of standard medium and finally replenished with 100 μl of fresh standard medium. 495-CD8^+^ T-cells (effectors) were rinsed with standard medium and added to the target cells at a 2:1 ratio and incubated for 24 hours. Following the incubation period, culture supernatants were collected and IFN-γ was measured using a commercial bovine IFN-γ ELISA kit in accordance with the manufacturer’s instructions (Mabtech, Stockholm, Sweden), and a FLUOstar OPTIMA ELISA plate reader (BMG LABTECH, Ortenberg, Germany). Supernatants containing IFN-γ concentrations exceeding the quantification range were reanalyzed following appropriate dilution using PBS and the final IFN-γ concentration was calculated.

For IFN-γ release assays in the presence of bacterial colonization, the EBL cells were infected with either wild-type *E. coli* O157:H7 (ZAP193) or the isogenic Δ*escN* T3SS mutant (ZAP2138) at an MOI of 100 and incubated in a humidified 5% CO_2_ atmosphere at 37°C for a total of 3 hours. EBL cells were subsequently washed three times with standard medium and then replenished with 100 μl of fresh standard medium before the addition of 495-CD8^+^ T-cells, as described above. Peptide pulsing, as described above, occurred either prior to or after bacterial colonization according to the experimental objectives.

### Statistical analysis

Data were analyzed using GraphPad Prism v9.0 (GraphPad Software, LLC).

## Results

### Establishing an *in vitro* model for *E. coli* colonization of bovine epithelial cells

To investigate which peptides are presented by MHCI on the surface of bovine epithelial cells colonized with *E. coli* O157:H7, we adopted an immunopeptidomic approach. A pre-requisite for these studies was establishing an *in vitro* model that enabled effective *E. coli* O157:H7 colonization, whilst preserving host-cell viability. To achieve this, the optimal MOI and period of colonization by *E. coli* O157:H7 of the EBL bovine epithelial cell line was determined. EBL cells were co-cultured with *E. coli* O157:H7 at an initial MOI of 10 or 100 for 3, 4, 6, and 7 hours. Fluorescence microscopy was used to assess EBL viability and enumerate the number of bacteria per EBL cell (Supplementary Figure S1). Extended culturing for 6–7 hours resulted in a ratio of 25–50 bacteria per EBL cell, but reduced cell viability, as assessed using Trypan Blue staining and a commercial apoptotic/necrotic kit (data not shown). An MOI of 100 for 3 hours yielded a bacteria/cell ratio of 10:1 without any measurable effect on cell viability. Confocal microscopy confirmed the presence of actin polymerization and A/E lesion formation (Supplementary Figure S2). As actin polymerization is dependent on a fully functional T3SS, successful Tir injection into the host cell cytoplasm and close bacterial attachment facilitated by the intimin-Tir complex, this indicated that these conditions permitted effective colonization.

### Defining the MHCI-genotype of the EBL cell line and the associated MHCI-peptide binding motifs

Using this optimized infection model, a tandem mass-spectrometry immunopeptidome profiling approach was employed to identify peptides presented by MHCI on EBL cells. EBL cells were prepared under three different test conditions: uncolonized (uninfected control), *E. coli* O157:H7 colonized (ZAP193) and co-culture with a *ΔescN* T3SS defective isogenic mutant (ZAP2138; which cannot colonize cells via the T3SS). A total of 12 683, 11 512, and 11 119 peptides were identified, respectively (Fig. 1a, left panel; Supplementary Table S1), with FDRs of 1.6%, 1.8%, and 1.9%. The vast majority of the immunopeptidome (>99.3% in all three samples) matched *Bos taurus* protein sequences. Consistent with MHCI-eluted peptides, the majority of these peptides had a length of between 8 and 11 amino acids, with 9-mers being the most common (Fig. 1a, right panel).

**Figure 1:**
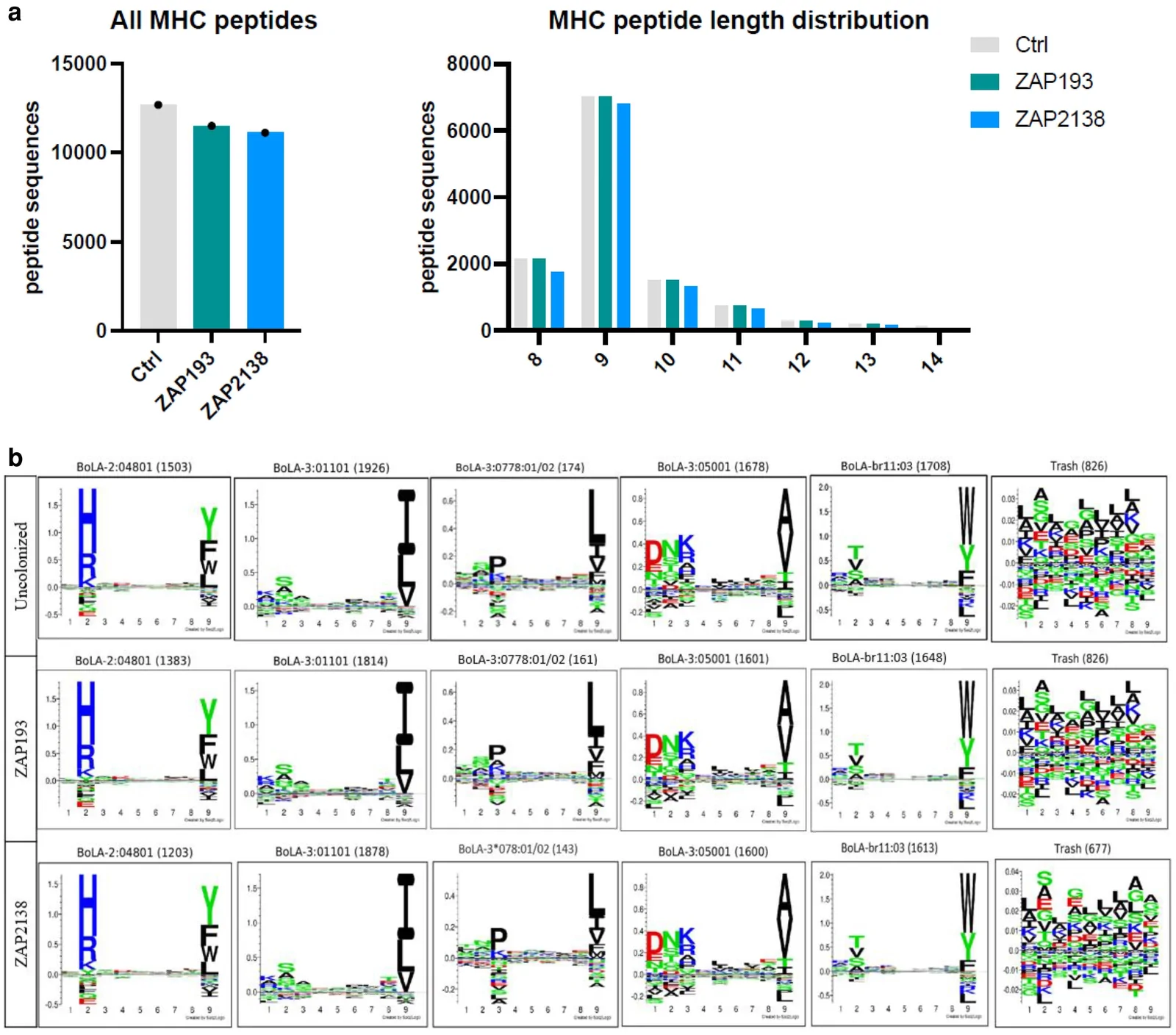
Immunopeptidome analysis of MHCI ligands eluted from EBL cells. (a) Summary of the immunopeptidomes derived from uncolonized (Ctrl), ZAP193 colonized and ZAP2138 co-cultured EBL. Left; number of peptides identified, right; length distribution of peptides. (b) Sequence motifs generated from peptides <14 amino acids from uncolonized, ZAP193-, and ZAP2138-colonized EBL cells using MHCMotifDecon-1.1 for each of the expressed MHCI alleles. Letter height reflects amino acid frequency at each position. The size of each letter is proportional to the number of peptides within each cluster sharing that amino acid at each position, with larger letters representing over-represented (above the x axis) or under-represented (below the x axis) amino acids. Peptides that appeared to not fit the peptide clusters were removed into a ‘trash’ bin. The name of each allele and the number of peptides used to form the motif are shown above each plot.

A pre-requisite for accurate MHC-binding prediction analysis of *E. coli* O157:H7-derived peptides was the defining of the MHCI alleles expressed by the EBL and the peptide-binding motifs of these alleles. MHCI (BoLA-I) genotyping of the EBL line (Supplementary Table S2) using an Illumina MiSeq-based protocol [29] identified the expression of 5 MHCI alleles: BoLA-3*011:01, BoLA-2*048:01, 3*078:01/02 (which together constitute the BF7 haplotype) and BoLA-3*050:01, and BoLA*br11:03, which together represent a variant of the HP1.60.1 haplotype described previously [30].

MHCMotifDecon-1.1 analysis using the *Bos taurus* protein-derived components of the immunopeptidome datasets identified the peptide-binding motifs of the individual MHCI alleles (Fig. 1b). Consistent with MHCI binding motifs, all five MHCI alleles had a strong preference for specific amino acids at the C’ terminus (position 9–P9), with specific preferences at the N terminus also observed (P2, and for some alleles P1 and P3 as well); as expected, these peptide-binding motifs were consistent across all three samples. Similarly, the proportion of peptides predicted to bind to each allele was consistent across the three samples (Supplementary Figure S3) and correlated with the percentage of reads ascribed to each allele in the MHCI-genotyping (Supplementary Table S2). For each allele the preferred length of binding peptides was nine amino acids (Supplementary Figure S3), with 8-mers also accommodated. For BoLA-2*048:01 and, to a lesser degree, BoLA*br11:03, there also appeared to be a greater tolerance for accommodating 10-mer and 11-mer peptides.

### Identification of *E. coli* O157:H7-derived MHCI ligands from colonized EBL cells

After preliminary refinement of the dataset (see Materials and methods and Supplementary Table S3), a total of 58 and 12 unambiguously *E. coli*-derived peptides were identified from the ZAP193 and ZAP2138(Δ*escN*) challenged EBLs (representing ∼0.5% and 0.1% of the total peptides sequenced) respectively. Notably, of the peptides identified in ZAP193-colonized cells, the majority (35/58, 60%) were derived from EspD. This protein forms part of the translocator apparatus of T3SS injection system and is integrated into the host cell membrane. The majority of these peptides (23/35, 66%) exceeded the canonical length of MHCI-binding peptides (i.e. were >13 amino acids in length) and formed clusters of nested, overlapping peptides in three distinct locations within the protein (Supplementary Figure S4). It is likely that the location of EspD in the host-cell membrane resulted in a collateral ‘pull-down’ of this protein during immunoprecipitation with peptides released during subsequent processing. Consequently, EspD-derived peptides were considered unlikely to represent genuine MHCI-presented peptides and were removed from the *E. coli* peptide dataset. This reduced the number of *E. coli* peptides in the ZAP193 dataset to 23, of which 10 were of the canonical length of MHCI-binders (i.e. 8–13 amino acids); similarly, removal of peptides with a length >13 amino acids reduced the number of peptides in the ZAP2138 (*ΔescN*) dataset to 11. Four of the peptides were identified in both the ZAP193 and ZAP2138 datasets, so the total number of unique *E. coli*-derived peptides identified was 17 (Table 1). Notably, only a single peptide was identified as being from a known translocated effector protein—EspF (Table 1).

**Table 1.** List of *E.coli*-derived peptides from samples ZAP193 and ZAP2138.

Peptide (A)	Sample (B)	Length (C)	#Spec (D)	Accession (E)	MHC (F)	BINDING_CORE (G)	RANK_SCORE (H)	RAW_SCORE (I)	Binder (J)
KAAPGQQI	ZAP193/2138	8	2	23293_ZAP193_02658 Property = intimin	BoLA-3:01101	KAA-PGQQI	0.008783184	0.794855595	Strong
ASSRLFAW	ZAP193/2138	8	1	23293_ZAP193_00780 Property = cytochrome c-type biogenesis protein CcmF	BoLA-br11:03	ASS-RLFAW	0.041556727	0.849758327	Strong
SASGLYGW	ZAP2138	8	1	23293_ZAP193_00175 Property = prepilin peptidase-dependent protein	BoLA-br11:03	SAS-GLYGW	0.179032713	0.633408546	Strong
KNDAAVLL	ZAP193	8	1	23293_ZAP193_03256 Property = YcaC—putative hydrolase	BoLA-3:01101	KN-DAAVLL	1.070557118	0.226223663	Weak
DQTKASITEI	ZAP193	10	1	23293_ZAP193_02658 Property = intimin	BoLA-3:05001	DQKASITEI	1.149100065	0.188229814	Weak
SSKVPALT	ZAP193	8	1	23293_ZAP193_00831 Property = putative HTH-type transcriptional regulatorRhmR	BoLA-3:05001	SSK-VPALT	2.002328396	0.132324338	Non-binder
TGGVKSVAL	ZAP193/2138	9	1	23293_ZAP193_04510 Property = acyl-coenzyme A dehydrogenase	BoLA-3:01101	TGGVKSVAL	2.087684631	0.136725828	Non-binder
PDSPALHVI	ZAP2138	9	1	23293_ZAP193_02137 Property = UvrABC system protein C	BoLA-3:01101	PDSPALHVI	4.004730225	0.071194358	Non-binder
TLANRLEI	ZAP2138	8	1	23293_ZAP193_04182 Property = urease accessory protein UreD	BoLA-3:01101	TLA-NRLEI	4.991509438	0.05493398	Non-binder
TLRQALNAT	ZAP2138	9	1	23293_ZAP193_02760 Property = exopolyphosphatase	BoLA-3:05001	TLRQALNAT	7.409196854	0.040522739	Non-binder
NDLIKLGV	ZAP2138	8	1	23293_ZAP193_04493 Property = sulfate-binding protein	BoLA-3:05001	NDL-IKLGV	7.439013958	0.040321533	Non-binder
ARQAPPPPTSG	ZAP193	11	1	23293_ZAP193_02667 Property = secreted effector protein EspF(U)	BoLA-3:01101	AAPPPPTSG	13.39836502	0.01193669	Non-binder
SGVSWGASAIV	ZAP193	11	1	23293_ZAP193_04281 Property = putative cystine transporter YijE	BoLA-3:01101	SSWGASAIV	14.48539162	0.01028978	Non-binder
PLQRALTV	ZAP193/2138	8	1	23293_ZAP193_04326 Property = TssM	BoLA-3:05001	PLQRALTV	17.85807228	0.012257223	Non-binder
YISGIGKDSVIWV	ZAP193	13	2	23293_ZAP193_02206 Property = Antigen Lp49	BoLA-br11:03	YISGIGKIW	30.79590607	0.000861123	Non-binder
APLMARAP	ZAP2138	8	1	23293_ZAP193_01857 Property = putative FAD-linked oxidoreductase Rv2280	BoLA-3:05001	APL-MARAP	33.59578705	0.003710053	Non-binder
CPIIENLSGCC	ZAP2138	11	2	23293_ZAP193_01333 Property = HTH-type transcriptional regulator CueR	BoLA-3:05001	CPIIENLSC	90.43287659	6.17967E-05	Non-binder

From each peptide the sequence (column A), sample(s) in which it was identified (B), the peptide length (C), the number of peptide-spectrum matches (#spec) (D) and the protein from which it was derived (E) are shown. Also shown are parameters associated with MHCI-binding prediction, with the MHCI allele exhibiting the highest predicted binding peptide score (F), the binding core of the peptide (G), rank score of predicted binding (H), raw score of predicted binding (I), and categorization of the binding prediction (J) shown. Peptides with rank scores of <0.5% were considered as strong binders, between 0.5 and 2% as weak binders and more than 2% as non-binders (K).

### MHCI ligands include peptides derived from intimin, the essential bacterial adhesion factor

Using the *Bos taurus*-derived peptides from the immunopeptidome datasets presented to train NetMHCpan (v4.1) permitted the development of bespoke algorithms to investigate the binding affinity of the *E. coli*-derived peptides for the MHCI molecules expressed by the EBL cell line *in silico.* The eluted ligand percentile rank score (EL_Rank) for each of the five BoLA alleles expressed by EBL cells was calculated for each of the 17 putative MHCI-ligands identified. The EL_Rank score provides a normalized prediction, permitting comparison between different MHCI alleles of the capacity of a peptide to bind, with EL_Rank scores of <0.5%, ≥0.5, and <2.0%, and ≥2.0% considered to indicate strong, weak, and non-binders, respectively. Only 5 of the 17 *E. coli*-derived peptides were predicted to be binders (three strong and two weak) to at least one of the alleles expressed by the EBL cells and hence considered to be probable bovine MHCI ligands (Table 1). Two of the strong-binding peptides, derived from intimin and cytochrome c-type biogenesis protein (CcmF), were identified in both ZAP193 and ZAP2138 datasets. An additional strong binder identified in ZAP2138 was derived from a prepilin peptidase-dependent protein, whilst in ZAP193, two weak binders were identified, originating from intimin and YcaC. Notably, all of the EspD-derived peptides which had been removed from the dataset as putative ‘collateral pulldown’ peptides had an EL_Rank >2%, supporting the notion that the peptides derived from this protein were not genuine MHCI-binders (Supplementary Table S3). The two intimin-derived peptides were presented by different MHCI alleles (BoLA-3*011:01 and BoLA-3*050:01 from the BF7 and HP1.60.1 haplotypes, respectively) and were derived from different parts of the intimin protein (Fig. 2). As such, intimin represented the main single-protein source of *E. coli*-derived peptides identified in the peptidome and predicted to be presented by MHCI molecules of EBL cells.

**Figure 2:**
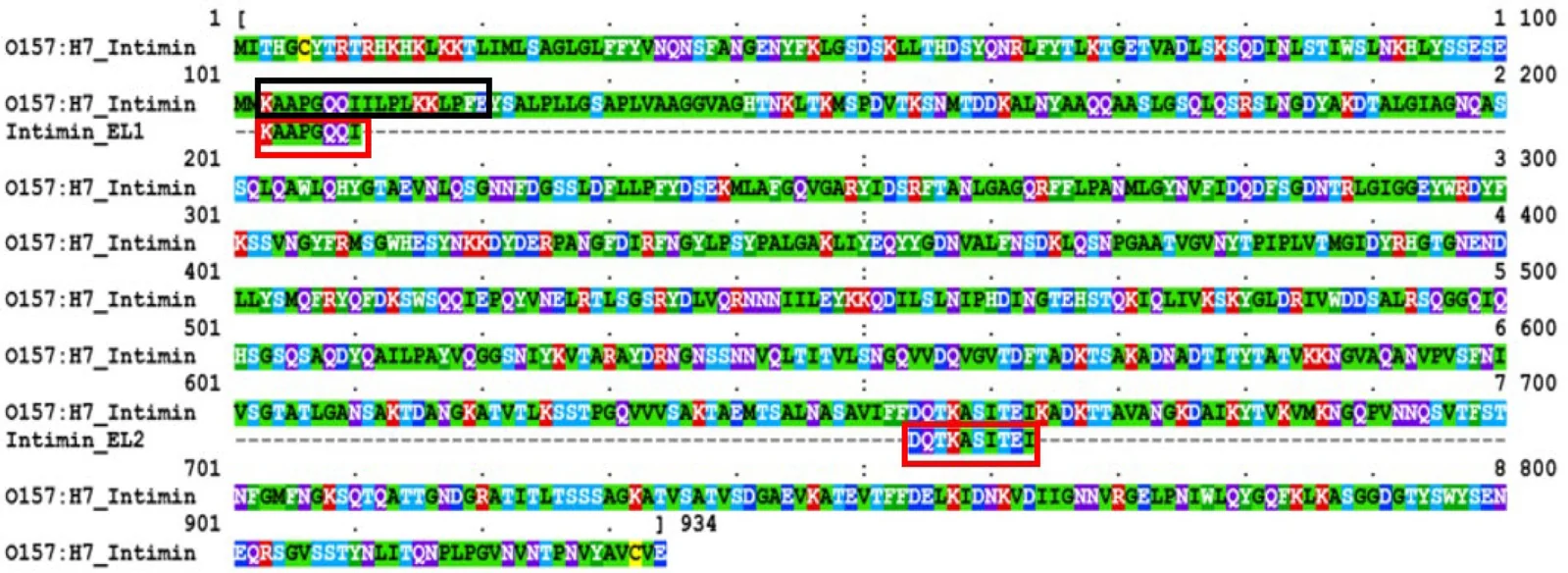
Graphical illustration of peptides eluted from the *E. coli* protein intimin, presented by BoLA-I molecules, following experimental colonization of a bovine epithelial cell line with *E. coli* O157. The figure shows the sequence alignment of putative BoLA-I ligands derived from the *E. coli* O157 protein intimin (NCBI sequence accession number 23293_ZAP193_02658). The black box indicates the alignment of the intimin peptide previously reported to provoke a CD4^+^ T-cell response [36], while the red boxes (EL_rank score <2%) highlight the alignment of intimin-BoLA-I ligands (EL1 KAAPGQQI coordinates 103–110 and EL2 DQTKASITEI coordinates 654–663), 8 and 10 amino acids in length, respectively, eluted in this study.

### BoLA-1*023:01 transfected EBL cells loaded with exogenous peptides efficiently activate antigen-specific CD8^+^ T-cells

Given the apparent dominance of structural extracellular bacterial proteins (i.e. non-translocated proteins) as a source of MHCI-presented peptides, we wanted to determine whether *E. coli* O157:H7-colonized bovine epithelial cells were able to stimulate antigen specific bovine CD8^+^ T cell responses to peptides processed via the exogenous MHCI pathway. Due to the limited reagents available for the study of bovine CD8^+^ T cell responses to *E. coli* O157:H7, and the absence of donor animals matched for any of the BoLA alleles expressed by EBL cells, we decided to develop an *in vitro* model utilizing available bovine cellular reagents (i.e. mono- and poly-clonal antigen-specific bovine CD8*^+^* T-cell lines of defined MHCI-restriction and epitope-specificity) developed for *T. parva* research. To facilitate this, EBL cells were transfected with the BoLA-1*023:01 allele, which is known to present the *T. parva* peptide Tp9­_67–75_ (AKFPGMKKS) [25] and can be identified using monoclonal antibody ILA89 (which does not bind any of the alleles endogenously expressed by EBL cells; Fig. 3a). A stable BoLA-1*023:01-expressing EBL cell line was generated by a combination of serial passage in selective medium and flow cytometric sorting using ILA89 (Fig. 3b). This line was pulsed with the Tp9_67–75_ peptide and used to stimulate a Tp9_67–75_/BoLA-1*023:01-specific CD8^+^ T-cell line (495-CD8^+^). 495-CD8^+^ T-cells demonstrated a dose-dependent IFNγ release following incubation with peptide-loaded BoLA-1*023:01-transfected EBL cells (Fig. 4a—red squares). The level of activation achieved by the Tp9_67–75_ peptide-pulsed BoLA-1*023:01-EBL was greater than that observed following equivalent stimulation of the 495-CD8^+^ T cell line using a Tp9_67–75_ peptide-pulsed immortalized peripheral blood mononuclear cell line endogenously expressing BoLA-1*023:01 (2229-Taa, BoLA-1*023:01-APC—orange triangles). Lack of T-cell activation following stimulation with the wild-type EBL cell line (WT EBL, not expressing BoLA-1*23:01—gray circles) pulsed with Tp9_67–75_ or BoLA-1*023:01^+^ cell lines pulsed with an irrelevant peptide (Tp2_98–106_; BoLA-1*023:01-EBL—green triangles and BoLA-1*023:01-APC—blue diamonds), confirmed stimulation of the T-cell line by the BoLA-1*023:01-transfected EBL cells was both MHC-restricted and peptide-specific.

**Figure 3:**
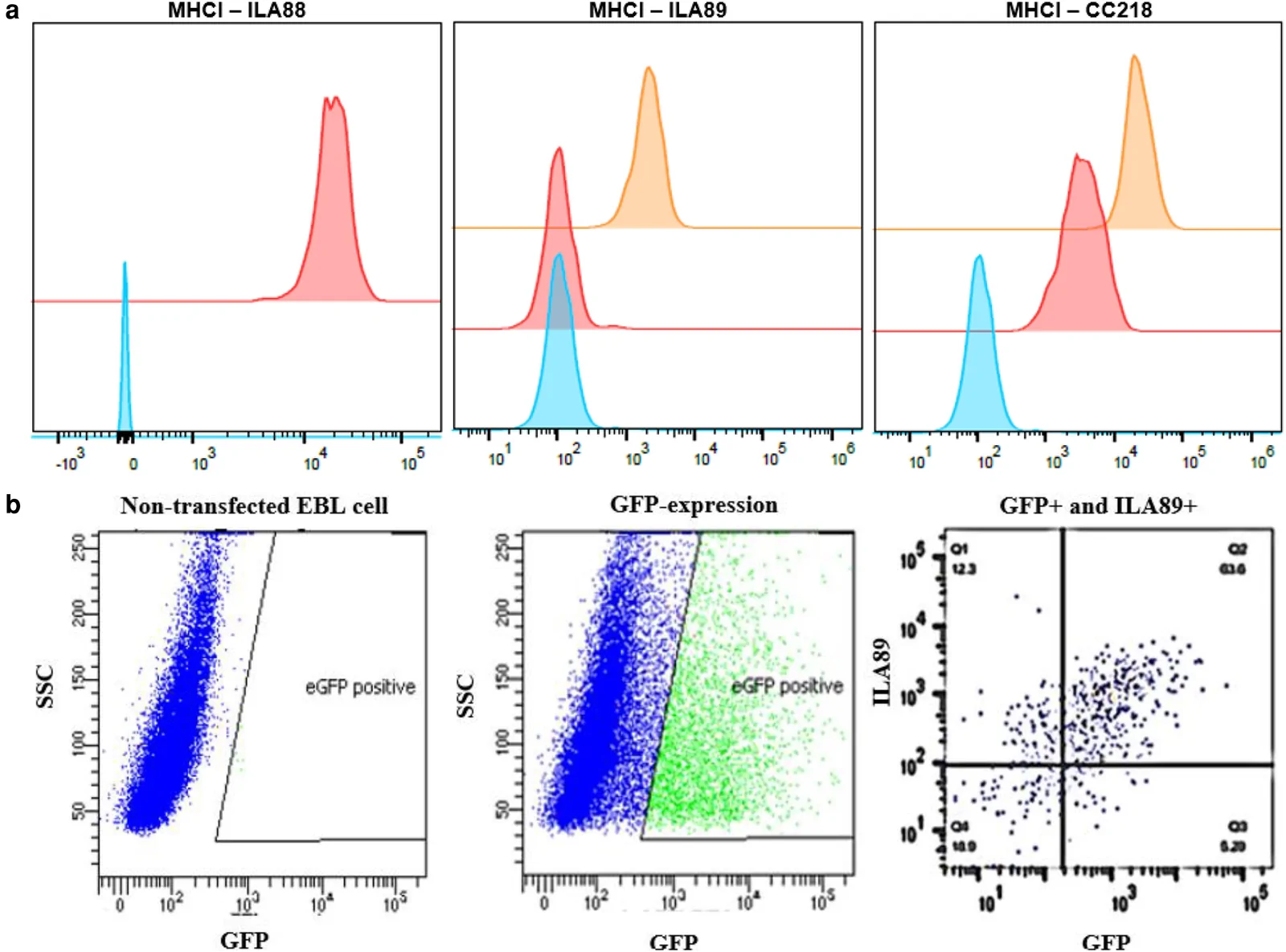
Generation and validation of BoLA-1*023:01 transfected EBL cell line. (a) MHCI expression profiling of parent EBL cells. Left panel: staining of parent EBL cell line with the ILA88 antibody (pan-MHCI antibody; red) or without antibody (negative control; blue); confirming expression of MHCI by the parent EBL cell line. Middle and right panels: staining with ILA89 and CC218 (polymorphic MHCI antibodies) respectively for parent EBL cell line (red), and 2229TpM, a BoLA-1*023:01 expressing *T. parva*-infected cell line (positive control for expression of BoLA-1*023:01; orange), with a control EBL staining with no primary antibody (blue). This demonstrated that ILA89, but not CC218 could be subsequently used for sorting BoLA-1*023:01-transfected EBL cell lines. **(**b**)** GFP expression and flow cytometric sorting of GFP^+^ and BoLA-1*023:01^+^ transfected cells (right panel, Q2) using ILA89.

**Figure 4:**
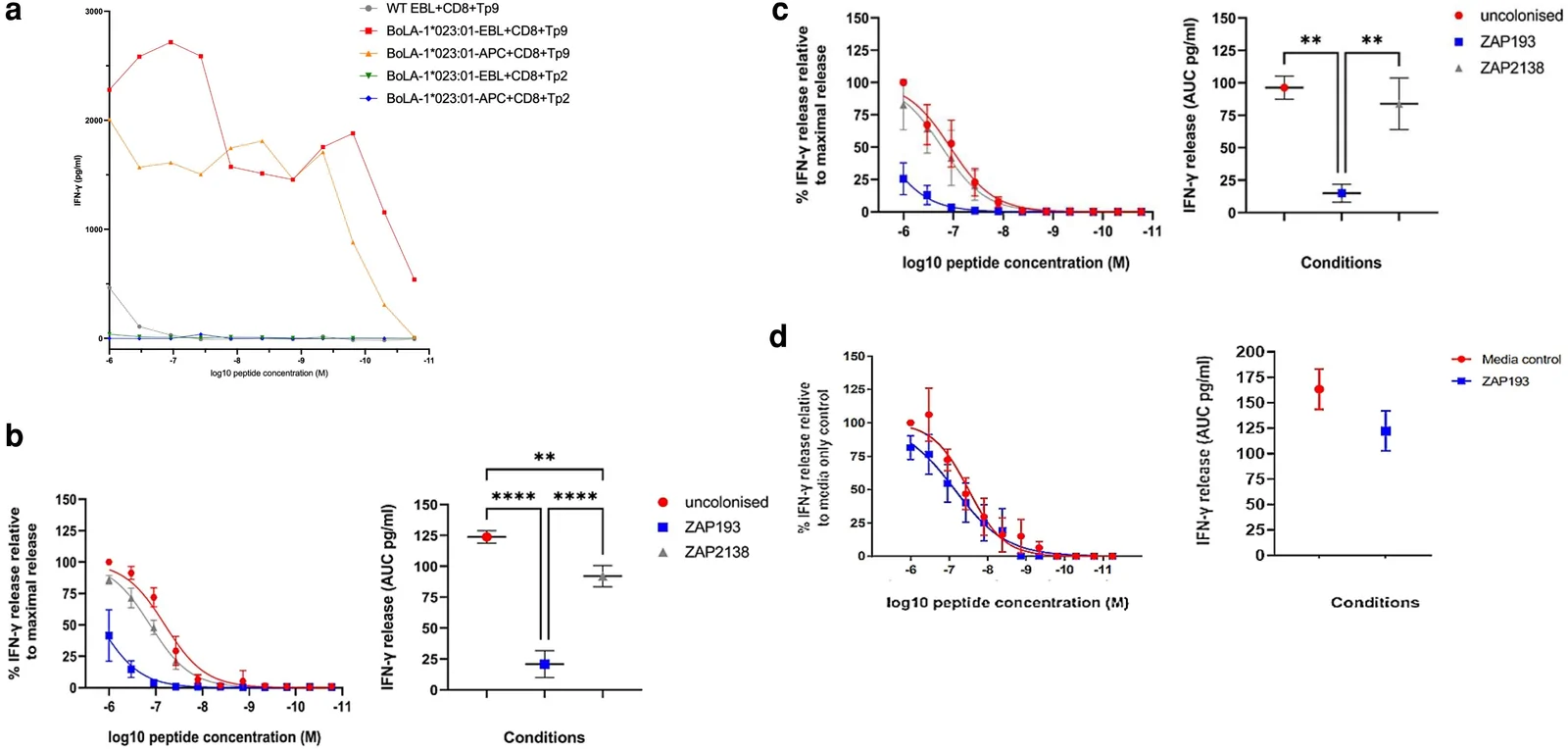
Functional validation of BoLA-1*023:01-transfected EBL cells and assessment of CD8^+^ T cell responses in the presence of *E. coli* O157:H7. (a) Validation of the BoLA-1*023:01 transfected EBL clone as a target cell line for antigen specific CD8^+^ T cells. IFN-γ release by a bovine CD8^+^ T cell line (495-CD8^+^) specific for Tp9_67–75_-BoLA-1*023:01 after 24 hours of co-culture with the following target cells: BoLA-1*023:01-EBL cells pulsed with Tp9_67–75_ (red squares), 2229-TaA, a BoLA-1*023:01 expressing *T. annulata*-infected cell line, cells pulsed with Tp9_67–75_ (orange triangles—positive control) and wild-type EBL cells pulsed with Tp9_67–75_ (gray circles—negative control). This confirmed the ability of the BoLA-1*023:01-EBL cells, but not the parent EBL to present epitopes on BoLA-1*023:01 to epitope-specific CD8^+^ T-cells. To confirm that this stimulation was epitope specific parallel assays using peptide pulsing with an alternative epitope (Tp2_98–106_) was completed; BoLA-1*023:01 transfected EBL cells pulsed with Tp2_98–106_ (green triangles), 2229-TaA cells pulsed with Tp2_98–106_ (blue diamonds); both negative controls. (b) Impact of bacterial colonization on CD8^+^ T cell stimulation (peptide pulsing after colonization): EBL cells were colonized with *E. coli* O157:H7 wild-type strain ZAP193 or the ΔescN T3SS-deficient mutant ZAP2138 prior to peptide pulsing with Tp9_67–75_. Left: IFN-γ release by 495-CD8^+^ T cells in response to increasing peptide concentrations. Right: area under the curve (AUC) analysis across four biological replicates. One-way ANOVA with Tukey’s multiple comparison test was used to determine statistical significance (*P < 0.05, **P < 0.01, ***P < 0.001, ****P < 0.0001). (c) Impact of bacterial colonization on CD8^+^ T cell stimulation (peptide pulsing before colonization): EBL cells were first pulsed with Tp9_67–75_, then colonized with ZAP193 or ZAP2138. Left and right panels as described in (b). (d) Impact of wild-type *E. coli* O157:H7 bacterial supernatant on CD8^+^ T cell stimulation using BoLA-1*023:01 transfected EBL cells as the target cell line. Left: IFN-γ release by 495-CD8^+^ T-cells as a proportion of maximal release in response to different Tp9_67–75_ peptide concentrations after incubation with WT- bacterial supernatants (blue square) in comparison to the medium control (red circle). Right: AUC for three technical replicates of each condition in the left panel. No statistically significance differences were observed between conditions, as determined by Student *t*-test *P < 0.05, **P < 0.01, ***P < 0.001, and ****P < 0.0001). ZAP193 = wild-type *E. coli* O157:H7 supernatant.

### *E. coli* O157:H7 colonization of bovine epithelial cells attenuates antigen-specific CD8^+^ T-cell activation

Having confirmed that antigen-pulsed BoLA-1*023:01^+^ EBL cells were able to stimulate antigen-specific IFNγ release by 495-CD8^+^ T-cells, the assay was repeated following EBL colonization with ZAP193 or co-culture with ZAP2138 bacteria at an MOI of 100 for 3 hours (Fig. 4b). Colonized cells were washed with antibiotic (penicillin and streptomycin)-containing medium prior to loading with Tp9_67–75_ and the subsequent addition of 495-CD8^+^ T-cells. There was a significant reduction in the capacity of ZAP193-colonized BoLA-1*023:01^+^ EBL cells to activate bovine CD8^+^ T-cells in comparison to uncolonized cells (*P* < 0.0001). Interestingly, this effect was reduced (*P* < 0.01), but not abolished (*P* < 0.0001), following co-culture with the *ΔescN* T3SS defective isogenic mutant ZAP2138. Together, these data indicate that *E. coli* O157:H7 has the capacity to reduce the effectiveness of peptide-MHCI dependent activation of CD8^+^ T-cells; however, this effect is much more substantial in bacteria with the capacity to colonize epithelial cells via the T3SS.

### Suppression of antigen specific CD8^+^ T cell activation is not a consequence of impaired exogenous peptide uptake, processing, and presentation by MHCI

To determine whether *E. coli* O157:H7 diminishes CD8^+^ T-cell activation via impairing exogenous peptide uptake, processing and presentation by MHCI, the assay was repeated using BoLA-1*023:01^+^ EBL cells that were pulsed with Tp9_67–75_ prior to colonization with ZAP193 or co-culture with ZAP2138. A reduction in antigen specific IFNγ release by 495-CD8^+^ T-cells (Fig. 4c) similar to that demonstrated in the previous experiment (Fig. 4b) was observed, indicating that suppression of T-cell activation was not a consequence of impairment of the exogenous peptide processing pathway, but due to a defect at the point of MHC-peptide presentation to the cognate TCR on the T-cell or some later stage of the T-cell activation pathway. Although it exhibited a similar trend, in contrast to the previous experiment, the reduction in IFNγ release by 495-CD8+ T-cells following co-culture with the T3SS-deficient ZAP2138 strain did not reach the threshold to be considered significant.

In a final experiment, the capacity of sterile ZAP193 culture supernatant to mediate inhibition was assessed by transferring 495-CD8^+^ T-cells into this supernatant for 16 hours prior to washing the T-cells, and co-culturing with Tp9_67–75_-pulsed BoLA-1*023:01^+^ EBL cells. No significant reduction in antigen-specific activation of 495-CD8^+^ T-cells was observed (Fig. 4d), indicating the inhibitory effect of the ZAP193 bacteria was either mediated as a consequence of colonization of the EBL cells or via a virulence factor that requires direct contact between the bacteria and CD8^+^ T-cells.

## Discussion

*Escherichia coli*, including *E. coli* O157:H7, are an important cause of foodborne bacterial infections, with high morbidity. Cattle are the dominant reservoir host for human infections. Vaccines that reduce bacterial load in cattle have the potential to reduce the incidence of human *E. coli* infections and the associated clinical consequences.

The longstanding approach to identifying immunologically relevant antigens of extracellular bacteria has primarily focused on antibody responses, with epitopes for CD4^+^ T cells to provide ‘help’ for B-cells also considered. Such strategies have dominated approaches to identifying antigens for most existing candidate vaccines targeting *E. coli* O157:H7. However, these vaccines have shown variable efficacy in reducing bacterial shedding and cattle-to-cattle transmission [18–22, 24–26]. Previous findings from our group have demonstrated robust responses by *E. coli* O157-specific CD8^+^, as well as CD4^+^, T cells, isolated from rectal lymph nodes of colonized calves, which drain the principal site of *E. coli* O157:H7 colonization in cattle [17]. Although *E. coli* O157:H7 is classified as an extracellular pathogen, it is capable of delivering multiple effector proteins into host cells via its T3SS; as such, it was hypothesized that, similar to Salmonella, from an antigen-presentation perspective, this could ‘mimic’ an intracellular infection and so lead to presentation of bacteria-derived proteins on host MHCI that subsequently elicit CD8^+^ T-cell responses [37, 38].

To test this hypothesis, we applied immunopeptidomics to an *in vitro* model of *E. coli* O157:H7 colonization of bovine epithelial cells. Recent studies have employed immunopeptidomics to identify MHC-presented peptides for a number of bacterial pathogens including *Listeria monocytogenes* [39], *Salmonella typhimurium* [40], and *Mycobacterium tuberculosis* [41–43], which in some cases has led to identification of antigens that successfully elicited protective immunity in model systems [39]. Such studies have supported the concept of using immunopeptidomics to accelerate candidate T-cell antigen identification, which remains a substantial hurdle in vaccine development for many bacterial pathogens.

Generation of robust bacterial immunopeptidomic datasets requires the availability of appropriate infection models and the implementation of quality control steps that can be implemented to filter the resulting data and so curate a ‘high-confidence’ list of MHCI-presented peptides. Consequently, there were two key pre-requisites for this study; (i) establishing a relevant *in vitro E. coli* O157:H7 infection model, in which the immunopeptidome of a bovine epithelial cell line (EBL) could be assayed after effective colonization and (ii) characterizing the MHC-genotype of the EBL cell line and defining the peptide-binding motif of the expressed BoLA-I alleles to enable *in silico* prediction of the capacity of the identified bacterial peptides to bind to the expressed MHCI. After accomplishing this, it was possible to analyze the *E. coli* O157:H7 immunopeptidome following culture of the EBL with two strains—one mainly colonizing via the T3SS (ZAP193, T3SS+) and an isogenic variant that cannot colonize via the T3SS (ZAP2138). A total of 70 unambiguously *E. coli*-derived peptides were identified from the two samples, representing about 0.3% of the total number of peptides identified in the total immunopeptidome. The low frequency of *E. coli* O157:H7 peptides is similar to the percentage of bacterial-origin peptides identified across a range of equivalent immunopeptidomic studies [44].

A large subset of the peptides from the ZAP193 sample were derived from EspD (35/58 peptides; 60%)—a bacterial protein that is part of the T3SS injection system which integrates into the host cell membrane. The EspD-derived peptides did not exhibit canonical features of MHCI-presented peptides, formed overlapping ‘clusters’ in discrete regions of the parent protein and so were considered to represent collateral ‘pull-down’ peptides resulting from isolation of membrane fragments during the immunoprecipitation preparation of samples. Peptides with similar features, that were considered to be ‘co-precipitated’, have been identified in other studies [45]. The presence of EspD peptides in the ZAP193, but not the ZAP2138, dataset was consistent with former, but not the latter, expressing a functional T3SS system, and provided evidence that the immunopeptidome data reflected the biological differences between the analyzed samples, as has been reported previously [46]. It was also interesting to note that there was an absence of peptides from EspB, considered a key component of EspADB translocon apparatus—suggesting that EspD, rather than EspB is the main bacterial protein that interacts directly with the host-cell membrane. Further refining of the *E. coli* dataset based on peptide length reduced the number of bacterial-derived peptides to 17, of which *in silico* predictions using the NetMHCpan4.1 algorithm [47], suggested only five were predicted to be MHCI-binding. Robust quality-control filtering of immunopeptidomic datasets to remove peptides that are considered unlikely to be genuine MHC-binders is essential for the generation of ‘high-confidence’ peptidomic datasets. Such curation is often dependent on a combination of (i) knowledge of the biological systems being investigated (e.g. host-cell membrane integration by EspD) and (ii) the application of filters based on known parameters of MHCI-binding peptides (e.g. peptide lengths and the specific peptide-binding motifs of expressed MHCI alleles).

The five *E. coli* peptides identified were derived from four proteins; intimin, CcmF, prepilin peptidase-dependent protein, and YcaC (a hydrolase). This list excludes peptides from any of the ∼40 effector proteins translocated by *E. coli* O157:H7 into the host cell cytosol, suggesting MHCI-presentation is instead dominated by exogenous antigens internalized by the epithelial cells. This was contrary to our initial hypothesis; there are a number of potential factors that may contribute to this observation. First, translocated effector proteins may be present at a low intracellular abundance relative to more readily processed proteins and/or have evolved to limit presentation on MHCI. Supporting the latter, epitope prediction algorithms [14] assign lower MHCI presentation scores to *E. coli* effector proteins compared to structural components, potentially indicating evolutionary adaptations to reduce epitope density or alter antigenic profiles. This mechanism may be analogous to viral escape mutations that disrupt MHCI presentation and prevent adaptive immune recognition [36, 48–50]. The presence of EspD peptides in the ZAP193 dataset and confirmation of actin polymerization by confocal microscopy during colonization permits the exclusion of a non-functional T3SS system as a cause of the absence of translocated protein-derived peptides in this system. Second, in addition to these biological considerations, the absolute number of *E. coli* peptides identified is low and likely to be far from ‘complete’ and higher resolution, more comprehensive immunopeptidomes may include peptides derived from translocated proteins. Regardless of these considerations, the data generated clearly supports exogenous *E. coli* proteins as a contributor to the MHCI-peptidome.

Of the five peptides identified, two (DQTKASITEI and KAAPGQQI) were derived from intimin, a 934-amino-acid transmembrane protein expressed on the surface of *E. coli* O157:H7 that binds to its translocated receptor (Tir) on the surface of the host cell. Identification of the KAAPGQQI peptide from the N-terminus of the protein, which was predicted to be a strong binder to the BoLA-3*011:01 allele, in the non-colonizing (ZAP2138) as well as the colonizing (ZAP193) *E. coli* O157:H7 sample suggests that MHCI-presentation was not dependent on direct T3SS-mediated association of the molecule with the host-cell surface via Tir. Similarly, absence of any Tir-derived peptides in the immunopeptidomes indicates that the intimin peptides are not present in the peptidome as a consequence ‘collateral pull-down’ of intimin-Tir complexes in the host-cell membrane. Together these observations support the processing and MHCI-presentation of intimin as an exogenous protein. The peptide DQTKASITEI (predicted to be a weak binder to the BoLA-3*005:01 allele) is located toward the C-terminus of the intimin molecule, within the bacterial immunoglobulin-like domain. In contrast, the KAAPGQQI peptide is located in the N-terminus of intimin, within the LysM domain, which is predicted to mediate protein binding to peptide glycan in the periplasm [51], and aligns with the previously reported CD4^+^ T cell epitope intimin 2.8 [52], which has been shown to elicit significant IFN-γ release from CD4^+^ T cells isolated from colonized calves. Due to its insolubility and purification difficulties, expression of full-length intimin has posed technical difficulties; consequently, most vaccine development studies have focused on C-terminal intimin fragments such as C280 [53], with particular interest in intimin-531 [25, 26]. However, since these exclude the N-terminal region harboring epitopes recognized by both CD4^+^ and CD8^+^ T cells [52], the potential immunogenicity of these constructs may be compromised and could be associated with sub-optimal protection in vaccinated animals [22, 25, 26].

Confirmation of MHCI-presentation of *E. coli*-derived peptides on epithelial cells supported the potential for CD8^+^ T-cells to have a role in mediating an anti-bacterial effect. *E. coli* O157:H7 colonization is critical for establishing persistent infections in cattle and recent work has proposed that *E. coli* Shiga toxin Stx2 sub-types may reduce epithelial cell turnover as a mechanism to sustain bacterial colonization [54]. Killing and subsequent removal of epithelial cells by *E. coli* O157:H7-specific CD8^+^ T-cells could inhibit colonization and thus facilitate clearance of infection. Generation of a BoLA-1*023:01^+^-EBL cell line permitted the development on an *in vitro* system to confirm the capacity of EBL cells to present an exogenously delivered peptide (the BoLA-1*023:01-restricted Tp9_67–75_ epitope from *T. parva*) to antigen-specific CD8^+^ T-cells. This system was subsequently utilized to explore the capacity of *E. coli* O157:H7 to inhibit CD8^+^ T-cell responses. The colonizing strain (ZAP193) was found to reduce IFN-γ release by responding CD8^+^ T-cells by ∼80%. This inhibitory effect was of similar magnitude if colonization of the epithelial cells occurred prior to, or after loading of, exogenous peptide, supporting that the inhibition was not disrupting peptide processing, but rather MHCI presentation or a post-presentation component of the T-cell activation pathway. The non-colonizing strain (ZAP2138), had a reduced but still significant effect, lowering CD8^+^ T-cell IFN-γ production by 25–30%, demonstrating that *E. coli* O157:H7 colonization via T3SS was a substantial factor of the inhibitory effect. Failure of ZAP193 culture supernatants to mediate CD8^+^ T-cell inhibition also supported the argument that either contact between the colonizing bacteria and host cells was required for effective inhibition or secreted effectors are impeding CD8^+^ T-cell activation via an effect on the epithelial cells. However, the mechanism by which this inhibitory effect is mediated remains to be determined. Potential options include disruption of immunological synapse formation between the CD8^+^ T-cell and its host-cell target, a virulence factor on the surface of the bacteria that mediates its inhibitory effect by direct contact with host cells (either the epithelial target or the CD8^+^ T-cell itself), or the creation by colonizing bacteria of a ‘micro-environment’ in which secreted factors reach a sufficient threshold to mediate an inhibitory effect, or some combination of these. A number of *E. coli* virulence factors have been demonstrated to inhibit T-cell responses, including Shiga toxins (Stx), lymphostatin (LifA), and the LiFA homolog Toxin-B (ToxB) [55–58]; however, the strain utilized in the current study does not encode Stx (through natural deletion) or LifA. Recent work on ToxB shows that while this factor can effectively inhibit T-cell proliferation, its deletion from *E. coli* did not abrogate this effect [58–60], and indicates redundancy in the *E. coli* O157:H7 mechanisms targeting T-cell function, supporting the biological significance of this inhibition. Consequently, detailed studies permitting effective discrimination between the different potential mechanisms will be required to elucidate which are operating to inhibit CD8^+^ T-cell activation in this system. Irrespective of the mechanisms employed, evidence that *E. coli* O157:H7 colonization is associated with inhibition of CD8^+^ T-cell function suggests that negating this arm of the immune response is biologically beneficial to the bacteria.

Collectively, our findings have (i) used immunopeptidomics to demonstrate that bovine epithelial cells can present *E. coli*-derived peptides on MHCI molecules and thus have the potential to be recognized by *E. coli*-specific CD8^+^ T-cells and (ii) demonstrated that colonizing *E. coli* O157:H7 are able to inhibit CD8^+^ T-cell function *in vitro*, suggesting subversion of this arm of the host immune response is of biological relevance to the bacteria. Together with previous data [52], our work supports the inclusion of MHCI-presented epitopes in future *E. coli* O157:H7 vaccines, to enable the recruitment of CD8^+^ T-cells in addition to the CD4^+^ T-cell and antibody responses that are the usual targets of *E. coli* O157:H7 vaccines. Our data support intimin as a good vaccine antigen candidate; immunopeptidomics demonstrates it is preferentially selected for MHCI-presentation and *in silico* predictions using NetMHCpan (v4.1) identifies intimin-derived peptides that can be presented across a broad range of BoLA-I alleles (data not shown); suggesting it would be an effective CD8^+^ T-cell antigen, complementing its already demonstrated capacity to function as both a CD4^+^ T-cell and B-cell antigen. Future studies should expand the immunopeptidomic dataset to epithelial cell lines expressing a diverse range of MHC-genotypes, and provide *in vitro* (and subsequently *in vivo*) validation of the candidate antigens thereby identified. Work to further understand *E. coli* O157:H7 inhibition of CD8^+^ T cells, the bacterial factors involved in this functional suppression and its implications for host-pathogen interactions would also be valuable. It is important to note that the work in this study used a *Stx*^−^ and *lifA*^−^ strain and so it is probable that *E. coli* O157:H7 field strains *in vivo* may possess redundant and/or synergistic immunosuppressive strategies. This is likely to have implications with respect to vaccine development, which may need to neutralize multiple virulence factors to achieve sterilizing immunity.

Rational approaches for T-cell antigen identification remains an obstacle for the development of vaccines needed to control a wide range of human and veterinary bacterial pathogens. Immunopeptidomics enables proteome wide, unbiased analysis of pathogen-derived MHC-presented peptides and is being increasingly employed to address this critical step in human vaccine development [44]. The application of immunopeptidomics to veterinary bacterial diseases is in its infancy, with only one previous study that the authors are aware of [61]. Expanding the application of immunopeptidomics to veterinary diseases has the potential to accelerate vaccine development with commensurate gains in animal health, welfare, and productivity as well as contributing to addressing anti-microbial resistance, a significant global ‘One Health’ challenge.

## Supplementary Material

kyag017_Supplementary_Data

## Data Availability

The original contributions presented in the study are included in the article. Further inquiries can be directed to the corresponding author.

## Permission to reproduce

Graphical abstract created in BioRender. Connelley, T. (2026) https://BioRender.com/b0c28ls.
