# Supplementary material for: E. coli O157:H7 evades CD8+ T cell immunity through T3SS-dependent suppression and limited MHCI presentation of translocated proteins
Source: Discov Immunol. 2026 Sep 9;5(1):kyag017. doi: 10.1093/discim/kyag017 (PMC13643489; doi:10.1093/discim/kyag017)
Supplement: Supplementary file 1 [file kyag017_supplementary_data.zip › Supplementary_Materials.docx]

Supplementary Material

Supplementary Data 1. ZAP193 proteome (5100 proteins) based on the annotated DNAseq data of *E. coli* O157:H7 EDL933 (taxid: 562, nr database-BLASTP)

**Supplementary Figure 1.** EHEC O157:H7 colonisation of EBL cells as assessed by fluorescent microscopy. EBL cells were incubated with EHEC O157:H7 at a multiplicity of infection (MOI) of 10 or 100 for 3, 4, 6, and 7 hours. Images were captured using a Zeiss StereoLumar V12 Stereo Fluorescence Microscope, 100x oil immersion lens and the 405, 488 and 543nm lasers. Cell nuclei (stained with Hoechst-33342 - blue), cell cytoskeleton (actin filaments stained with phalloidin-FITC - green), and *E. coli* (stained with polyclonal anti-*E. coli* O157 specific antibody with a secondary Alexa-flour 568 conjugated antibody – red) are shown (co-localisation with other fluorescently labelled targets results in many *E. coli* appearing yellow/white). Images analysed using ImageJ.

**Supplementary Figure 2.** EHEC O157:H7 colonisation of EBL cells at a multiplicity of infection (MOI) of 100 for 3 hours as assessed by confocal microscopy. Image captured using a Zeiss LSM-880 confocal microscope with a Plan Apochromat x63/1.4NA lens and 405, 488 and 543nm lasers. Cell nuclei (marked with black asterisks, stained with DAPI – note that due to overlaying of other fluorescent labelled targets, the nuclei are not shown as blue), cell cytoskeleton (actin filaments stained with phalloidin-FITC – green), and *E. coli* (stained with polyclonal anti-*E. coli* O157 specific antibody with a secondary Alexa-flour 568 conjugated antibody – red) are shown. Image processing and presentation used Imaris 9.03 software. The image demonstrates bacterial attachment to the epithelial surface, with clear localised polymerisation of the actin cytoskeleton (white arrows).

*****

**Supplementary Table 1: Peptides identified from tandem mass-spectrometry analysis of a) control EBL, ii) ZAP2138 co-cultured EBL and iii) ZAP193-colonised EBL.** For each peptide the amino acid sequence (column A), -10lgP score (B), mass (C), peptide length (D), ppm (E), m/z (F), RT (G), intensity (H), Fraction (I), scan (J), source file (K), number of spectra detected (L), the identity of the protein from which it was derived (M), post-translation modification (N) and AScore (O) are shown.

**Supplementary Table 2: MHCI genotyping of EBL cell line.** The MHCI alleles expressed by the EBL cell-line were studied using a MiSeq-based high-throughput sequencing approach. Samples from the cell-line were analyzed as 3 independent replicates. The data presented here represents the average values derived from these 3 replicates. For each allele (column A) the assigned haplotype (B), the average read count (C), the percentage of reads assigned to each allele (D) and the normalized percentage of reads (E) assigned to each allele across the 3 repeats is shown. The percentage of read counts is ~72% as a subset of reads are discarded during QC of the data (e.g. poor-quality reads, read clusters below pipeline thresholds and chimeras); this is accounted for in the normalized percentage of read counts (where percentage is calculated only on reads assigned to individual MHCI alleles). Notes (column F) * - reads assigned to BoLA-3*078:01/02 are matches for both of these alleles as the SNP that distinguishes these alleles lies outwith the area amplified and sequenced in this protocol; hence the designation is ambiguous; ** reads assigned as BoLA-br11:03 appear to be variants of an allele previously identified in a Brazilian cohort (30).

**Supplementary Figure 3:** Demonstrates the length and count distribution of the peptides eluted from BoLA-I (five alleles) expressed on the surface of the EBL cell line, generated by MHCMotifDecon-1.1. X-axis represents the peptide amino acids length, while the y-axis represents the number of peptides eluted. Conditions denoted as (A) Control (uncolonized EBL), (B) EBLs colonised by ZAP193, and (C) EBLs cultured with ZAP2138.

**Supplementary Table 3: Unambiguous *E. coli*-derived peptides identified in the immunopeptidome from i) control EBL, ii) ZAP2138 co-cultured EBL and iii) ZAP193-colonized EBL.** For each peptide the amino acid sequence (column A), -10lgP score (B), mass (C), peptide length (D), ppm (E), m/z (F), RT (G), intensity (H), Fraction (I), scan (J), source file (K), number of spectra detected (L), the identity of the protein from which it was derived (M), post-translation modification (N), AScore (O), peptides derived from EspD (P) and peptides longer than the canonical MHCI-binding length (Q) are shown. Also shown are parameters associated with MHCI-binding prediction, with the MHCI allele exhibiting the highest predicted binding peptide score (R), the binding core of the peptide (S), rank score of predicted binding (T), raw score of predicted binding (U) and categorization of the binding prediction (V) shown. PTM peptides are shown but not included in the total of 47 peptides identified from the ZAP193 sample (for each an identical, non-PTM peptide was also identified in the ZAP193 sample)

**Supplementary Figure 4 - Peptides derived from EspD protein in the ZAP193-colonised sample**. EspD is a component of the T3SS injection system that integrates into the host cell membrane. It is 374 amino acids long and has 2 transmembrane domains (TM shown in red). A total of 24 peptides were identified as being derived from the EspD protein (an additional 11 post-translationally modified (PTM) peptides from EspD were identified in the ZAP193 sample (See Supplementary Table 2)). The 24 peptides from EspD represented nested, overlapping peptides derived from 3 distinct locations in the protein outside of the transmembrane domain: peptide cluster 1 (71-86aa), peptide cluster 2 (134-159 aa) and peptide cluster 3 (314-374aa). The majority of EspD-derived peptides were >13 amino acid in length (average of 17.0aa, Supplementary Table 3). This profile suggests the peptides derived from this protein may be present in the dataset as a consequence of collateral 'pull down' during the MHCI-immunoprecipitation - consistent with the location of the EspD protein in the host cell membrane. It is therefore unlikely they represent genuine MHCI-binding peptides and thus these peptides were removed from the *E. coli* peptide dataset (Table 1).
